# Unraveling the dual role of METTL3-mediated m^6^A RNA modification in bladder cancer: mechanisms, therapeutic vulnerabilities, and clinical implications

**DOI:** 10.1080/15384047.2025.2545057

**Published:** 2025-08-08

**Authors:** Hua Chun, Kangzhuo Baima

**Affiliations:** College of Medicine, Tibet University, Tibet, China

**Keywords:** Bladder cancer, METTL3, RNA m^6^A modification, epitranscriptomics, chemoresistance, immune evasion, therapeutic targeting

## Abstract

Bladder cancer (BC) remains challenging due to its recurrence and metastasis, with METTL3-mediated m^6^ A RNA modification emerging as a key oncogenic driver. This review synthesizes METTL3’s roles in BC progression, including tumor initiation, metastasis, stemness, and therapy resistance. We detail its regulation of critical pathways (e.g. HIF1A/IGF2BP3/BIRC5, AFF4/NF-κB/c-MYC) and dual functions in RNA stability and epigenetic crosstalk with DNA methylation. METTL3 promotes chemoresistance (e.g. circ0008399/WTAP/TNFAIP3) and immune evasion (PD-L1 stabilization), while its overexpression correlates with poor prognosis and cisplatin resistance. By integrating METTL3’s interactions with m^6^ A readers (YTHDF1/2, IGF2BP3) and erasers (ALKBH5), we propose targeting METTL3 as a strategy to enhance chemotherapy and immunotherapy efficacy. This work underscores METTL3’s potential as a diagnostic biomarker and therapeutic target, advancing precision oncology in BC.

## 1. Introduction: bladder cancer and the emerging role of METTL3-mediated epitranscriptomics

Bladder cancer (BC) ranks 9th in global incidence, with mortality projected to increase by 30% in low-resource regions by 2040.^1^ A 2023 meta-analysis confirms recurrence rates exceed 70% despite multimodal therapy,^2^ while global burden studies reveal an 85% incidence surge in aging populations from 1990–2021BC remains a critical public health challenge.^3^ The pathogenesis of BC is intricately linked to tumor risk genes,^3^ making it imperative to explore the post-transcriptional regulation of gene expression for a comprehensive understanding of BC pathogenesis.^4^

N^6^-methyladenosine (m^6^A) is a prevalent internal modification that plays a crucial role in gene expression post-transcriptionally across various RNA types, including mRNAs, lncRNAs, circRNAs, snRNAs, tRNAs, and ribosomal RNAs.^5,6^ The dynamic nature of RNA m^6^A modification relies on distinct functional components known as the writer, eraser, and reader.^7,8^ The writer complex primarily consists of methyltransferase 3/14(METTL3/14) and Wilms’ tumor 1‑associating protein (WTAP), which catalyze the target RNAs through the METTL3/METTL14/WTAP complex formation.^9^ The eraser function is carried out by FTO and ALKBH5, responsible for removing RNA methylation marks.^10^ On the other hand, readers encompass methyl recognition proteins such as YTH N^6^-methyladenosine RNA binding protein family (YTHDF), YTH domain-containing protein family (YTHDC),^11^ insulin-like growth factor 2 mRNA-binding protein family (IGF2BP),^12^ along with additional readers like HNRNPC, HNRNPG, and HNRNPA2B1.^13^ Consequently, m^6^A methylation governs diverse aspects of RNA processing including alternative splicing events, translocation processes as well as stability and translation mechanisms thereby influencing multiple biological processes.^14,15^

METTL3 serves as the central component of the m^6^A methyltransferase complex.^16^ Primarily, METTL3 governs the methylation of adenine molecules by modulating the translation of specific genes present in post-transcriptional RNAs.^17^ An investigation has demonstrated that aberrant m^6^A modification mediated by METTL3 is intimately associated with the onset, progression, and advancement of BC, thereby regulating malignant functions encompassing tumor cell proliferation, invasion, metastasis, and drug resistance.^18^ However, a comprehensive review summarizing these studies and evaluating these associations remains lacking.

This review synthesized evidence from PubMed, Web of Science, and EMBASE databases (2010–2025) using keywords: ‘METTL3,’ ‘m^6^A,’ ‘bladder cancer,’ ‘chemoresistance,’ ‘immunotherapy.’ Inclusion criteria^1^: Original studies focusing on METTL3 in BC^2^; Mechanistic/clinical validation *in vitro*/*in vivo*^3^; English-language publications. Excluded: Case reports, non-peer-reviewed articles. Data extraction prioritized consensus findings from ≥3 independent studies.

This review comprehensively assesses the roles, mechanisms, and clinical potential of METTL3 in BC, meticulously examining research progress, elucidating the correlation between METTL3 and BC, and providing valuable insights for future investigations as well as potential targets for clinical diagnosis, treatment, and prognosis. We conceptualize METTL3‘s ‘dual role’ as^1^: an oncogenic driver that accelerates bladder carcinogenesis through m^6^ A-dependent RNA remodeling of tumor-promoting pathways, and^2^ a therapeutic vulnerability whose inhibition sensitizes tumors to conventional and immune-based therapies. This duality positions METTL3 as both a disease amplifier and actionable target in precision oncology.

## 2. METTL3: a multifaceted orchestrator of BC pathogenesis

BC is a prevalent malignancy of the urogenital system, characterized by high heterogeneity and recurrence rates, as well as a propensity to metastasize, leading to low survival rates and poor overall prognosis.^19^ However, the pathogenesis of BC is multifaceted and remains largely unclear. METTL3 plays a pivotal role in BC’s growth, progression, malignancy, and chemosensitivity by directly and indirectly regulating the translation and degradation of specific genes. Therefore, elucidating the mechanism underlying BC onset and progression from the perspective of METTL3 has become a major research focus.

### 2.1. METTL3 as oncogenic driver: from tumor initiation to metastasis

The onset and progression of BC are typically triggered by environmental risk factors, which collaborate with tumor-associated genes regulated by METTL3-mediated m^6^A modification.^20^ As a risk factor, fine particulate matter (PM2.5) acts as an inducer for BC.

#### 2.1.1. Environmental carcinogens and METTL3 synergy: epigenetic drivers of BC initiation

**Figure 1.**
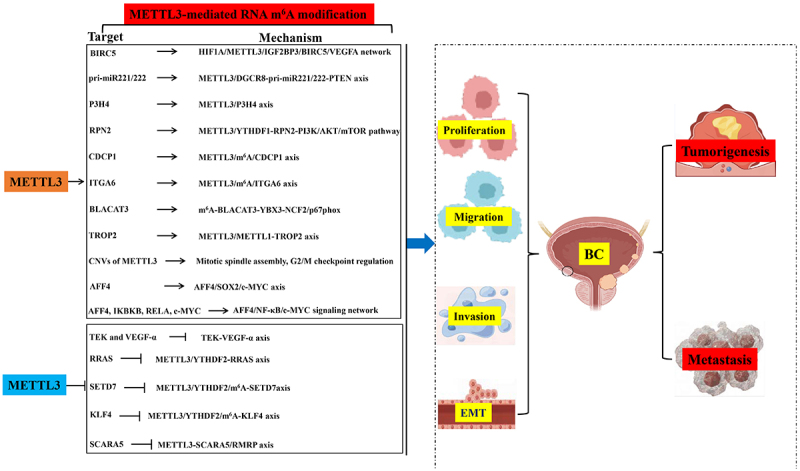
METTL3-centric network driving BC pathogenesis.

As reported, long-term exposure to PM2.5 induces BC. Yuan et al.^21^ reported aberrant upregulation of METTL3 expression after PM2.5 exposure, which dramatically enhances BC proliferation, colony formation, migration, and invasion while repressing apoptosis and disrupting the cell cycle. Mechanistically, PM2.5 enhances METTL3 expression by inducing its promoter hypomethylation and increasing its binding affinity to the transcription factor *HIF1A*.^21^ Additionally, *BIRC5* is an oncogenic gene that has been implicated in various human cancers.^22^ Exposure to PM2.5 enhances the m^6^A modification on *BIRC5*, leading to increased binding of IGF2BP3 to the 3’-untranslated region (3’-UTR) of *BIRC5* and ultimately upregulating its expression. This subsequently triggers PM2.5-induced angiogenesis by regulating VEGFA expression.^21^ These findings suggest that exposure to PM2.5 triggers the initiation and progression of BC by targeting the HIF1A/METTL3/IGF2BP3/BIRC5/VEGFA network, emphasizing the crucial role of METTL3 in regulating tumor genes. Additionally, METTL3 exerts oncogenic effects in BC through its interaction with the microprocessor protein DGCR8, promoting pri-miR221/222 maturation and subsequently reducing PTEN levels, thereby facilitating BC proliferation.^23^ Similarly, Prolyl 3-hydroxylase family member 4 (P3H4) is significantly overexpressed and strongly associated with BC onset and progression by enhancing cell proliferation, migration, invasion, and epithelial–mesenchymal transition (EMT).^24^ Liu et al.^25^ demonstrated that the upregulation of P3H4 is primarily attributed to METTL3-mediated m^6^A modification which regulates *P3H4* mRNA stability in BC. Furthermore, inhibition of METTL3 suppresses BC growth and EMT by targeting *P3H4* directly. Notably, METTL3‘s environmental (PM2.5→BIRC5) vs. intrinsic (P3H4 stabilization) activation mechanisms reveal context-dependent oncogenicity – a distinction with therapeutic implications for geographically tailored interventions. Collectively, these findings highlight the potential therapeutic significance of targeting the METTL3/P3H4 axis for treating BC (Figure 1, Table 1).

As the core m6 A methyltransferase, METTL3 orchestrates BC pathogenesis by dynamically regulating RNA stability, translation, and degradation differently. This figure integrates molecular mechanisms of METTL3’s function in BC, highlighting METTL3 as a disease driver in BC. Arrows denote activation; blunt lines inhibition.

METTL3, Methyltransferase-like 3; BC, bladder cancer; m6A, N6-methyladenosine; IGF2BP, Insulin-like growth factor 2 mRNA-binding proteins; VEGF-α, Vascular endothelial growth factor-α; PTEN, Phosphatase and tensin homolog; P3H4, Prolyl 3-hydroxylase family member 4; ITGA6, Integrin subunit alpha 6; BLACAT3, BLC-associated transcript 3; TROP2, Trophoblast cell surface protein 2; SETD7, SET domain-containing 7; KLF4, Krüppel-like factor 4; CNVs, Copy number variation; AFF4, ALF transcription elongation factor 4; SOX2, SRY-box transcription factor 2; c-MYC, Cellular MYC; TEK, Tyrosine endothelial kinase; IKBKB, Inhibitor of NF-κB; RELA, RELA proto-oncogene; EMT, epithelial–mesenchymal transition.

**Table 1.** METTL3-regulated molecular targets: functional landmarks and critical analysis in bladder cancer progression.

Category	Target	Function (Direction)	Biological Impact	Critical Synthesis	Ref
**Proliferation**	*BIRC5*	Up ↑ (IGF2BP3 binding)	PM2.5-induced angiogenesis	Limited *in vivo* validation of PM2.5-BIRC5 axis; clinical relevance unclear	^21^
	miR221 /222	Up ↑ (pri-miRNA maturation)	PTEN suppression	Tissue-specific effects; lacks comparison with other miRNAs	^23^
	*P3H4*	Up ↑ (mRNA stability)	EMT promotion	Contrasts with METTL3‘s environmental triggers (e.g., PM2.5)	^25^
	*RPN2*	Up↑ (YTHDF1-mediated)	PI3K/AKT/mTOR activation	*In vitro* only; clinical correlation missing	^26^
**Metastasis** **/EMT**	*CDCP1*	Up ↑ (YTHDF1 translation)	Chemical carcinogenesis	Overlaps with ALKBH5 regulation; demethylase crosstalk unexplored	^27,28^
	*ITGA6*	Up ↑ (YTHDF1/3 translation)	Cell adhesion/ invasion	Therapeutic targeting demonstrated; lacks *in vivo* metastasis data	^29^
	*SETD7*	Down ↓ (YTHDF2 degradation)	Tumor suppression loss	No clinical prognostic correlation reported	^30^
	*KLF4*	Down↓ (YTHDF2 degradation)	Tumor suppression loss	Limited to cell lines; *in vivo* relevance unknown	^30^
	CLASP2 /IQGAP1	Up↑ (TNF-α/m^6^A axis)	Cytoskeleton remodeling	Novel 2025 study; requires independent validation	^31^
**Stemness**	*AFF4*	Up ↑ (m^6^A stabilization)	SOX2/c-MYC transcriptional activation	Overlaps with NF-κB pathway; context-specific effects unaddressed	^32,33^
	*c-MYC*	Up↑ (AFF4-mediated)	BCSC self-renewal	Conflicting reports in other cancers	^33^
**Angiogenesis**	*VEGFA*	Up↑ (BIRC5-mediated)	PM2.5-induced angiogenesis	Indirect METTL3 regulation; direct m^6^A sites unmapped	^21^
	*TEK*	Down ↓ (m^6^A reduction)	Tumor angiogenesis suppression	Mouse model only; human tissue validation limited	^34^
**Chemoresistance**	*TNFAIP3*	Up ↑ (circ0008399 complex)	NF-κB pathway inhibition	No clinical trial data on targeting this axis	^35^
**Immune Evasion**	PD-L1	Up ↑ (IGF2BP1 stabilization)	CD8+ T-cell suppression	JNK crosstalk may vary by molecular subtype	^36^
**Epigenetic Crosstalk**	*TROP2*	Up ↑ (dual m^6^A/m7G)	Tumor progression	METTL1 codependency; single-target inhibition ineffective	^37^
	*SCARA5*	Down ↓ (RMRP-mediated methylation)	Tumor suppression loss	DNA methylation focus; RNA demethylase role unexplored	^38^
**Prognosis**	*METTL3*	Up ↑ (CNVs/ overexpression)	Poor OS	Paradox: CNVs correlate with chemo-sensitivity	^39,40^

METTL3, Methyltransferase-like 3; m6A, N6-methyladenosine; IGF2BP, Insulin-like growth factor 2 mRNA-binding proteins; VEGF-α, Vascular endothelial growth factor-α; PTEN, Phosphatase and tensin homolog; P3H4, Prolyl 3-hydroxylase family member 4; ITGA6, Integrin subunit alpha 6; BLACAT3, BLC-associated transcript 3; TROP2, Trophoblast cell surface protein 2; SETD7, SET domain-containing 7; KLF4, Krüppel-like factor 4; CNVs, Copy number variation; AFF4, ALF transcription elongation factor 4; SOX2, SRY-box transcription factor 2; c-MYC, Cellular MYC; TEK, Tyrosine endothelial kinase; IKBKB, Inhibitor of NF-κB; RELA, RELA proto-oncogene.

#### 2.1.2. METTL3/YTHDF1 axis fuels malignant transformation via PI3K/AKT/mTOR signaling

Building on METTL3’s synergy with environmental carcinogens, we next dissect its coordination of PI3K/AKT signaling – a nodal pathway bridging proliferation to therapy resistance. METTL3 interacts with m^6^A readers to regulate the expression of target genes. For instance, RPN2 plays a role in regulating BC proliferation, invasion, and drug resistance.^41^ Zhu et al.^26^ discovered that the METTL3/YTHDF1 axis promotes BC cell proliferation and inhibits *Cisplatin* sensitivity by mediating m^6^A modification on *RPN2* mRNA, which subsequently activates the PI3K/AKT/mTOR pathway. Notably, METTL3’s regulation of invasion extends beyond PI3K/AKT. The following section examines its direct control of metastatic RNA targets. Studies have investigated the dynamic modification of m^6^A mRNA during cellular transformation induced by chemical carcinogens and identified a subset of consistently modulated m^6^A sites associated with cell transformation.^27,28^ Importantly, malignancy transition enhances m^6^A modifications within the 3’-UTR of the *CDCP1* mRNA (an oncogene) through the recognition of YTHDF1 and thereby promoting CDCP1 translation.^27^ Mechanistically, this modification is mediated by METTL3 and m^6^A demethylases ALKBH5.^27^ The expression levels of METTL3 and CDCP1 are upregulated in BC patient samples and positively correlated with disease progression.^28^ The synergistic action of the METTL3/m^6^A/CDCP1 axis with chemical carcinogens promotes malignant transformation of uroepithelial cells as well as BC tumorigenesis *in vitro* and *in vivo*.^28^ Moreover, inhibition of the METTL3/m^6^A/CDCP1 axis reduces chemosensitivity while promoting BC growth and progression. These findings collectively demonstrate dynamic m^6^A modifications during chemically induced malignancy transitions and provide insights into the critical role played by the METTL3/m^6^A/CDCP1 axis in chemical carcinogenesis. Consistent with the findings of this study, upregulation of METTL3 expression significantly suppresses RRAS expression by enhancing its m^6^A modification level, which is governed by YTHDF2 binding to the m^6^A modification site on *RRAS* mRNA and subsequently inducing its degradation.^42^ Importantly, METTL3 plays a pivotal role in the malignant progression of BC through modulation of the RRAS/YTHDF2 signaling axis, thereby facilitating cell proliferation, migration, and invasion (Figure 1, Table 1).

#### 2.1.3. METTL3-mediated m^6^A dynamics: paving the way for BC metastasis

Metastasis contributes to the malignancy of BC, involving the participation of METTL3, which promotes cancer cell metastasis both *in vitro* and *in vivo*.^30^ For example, the METTL3/YTHDF2/m^6^A axis directly degrades two tumor suppressor mRNAs, SET domain-containing 7 (*SETD7*) and Krüppel-like factor 4 (*KLF4*), thereby contributing to BC progression.^30^ Conversely, overexpression of SETD7 and KLF4 leads to a phenotype consistent with depletion of the m^6^A axis. Other study suggests that METTL3 and ALKBH5 regulate cell adhesion by modulating integrin subunit alpha 6 (ITGA6) expression in BC cells.^29^ The *ITGA6* transcript is highly enriched with m^6^A modification and increased m^6^A modification on the 3’-UTR of *ITGA6* mRNA, enhances *ITGA6* translation through binding to YTHDF1 and YTHDF3 (m^6^A readers).^29^ Regulation of *ITGA6* partially restores adhesion, migration, and invasion abilities of BC cells along with elevated METTL3 expression in human BC tissues and lower patient survival rates. Conversely, inhibition of ITGA6 slows down the growth and progression of BC cells both *in vitro* and *in vivo*. In line with these findings, Ying et al.^43^ reported therapeutic potential targeting multisite m^6^A modifications on *ITGA6* in BC. Furthermore, bladder cancer-associated transcript 3 (BLACAT3) exhibits high expression levels in BC tissues positively correlated with poor prognosis among patients with muscle-invasive BC.^44^ Xie et al.^44^ demonstrated that upregulated BLACAT3 levels are strongly associated with m^6^A modification which stabilizes *BLACAT3* structure. Subsequently, overexpressed BLACAT3 regulates NCF2/p67phox expression by interacting with RNA-binding protein YBX3 to mediate angiogenesis and hematogenous metastasis in BC.^44^ Nonetheless, this investigation did not clarify the specific regulator responsible for mediating the m^6^A modification on *BLACAT3*, necessitating further exploration (Figure 1, Table 1).

METTL3, through mechanisms reliant on m^6^A readers, orchestrates the oncogenic activities of tumor-associated genes. This coordination aids in the initiation, progression, and metastasis of BC by regulating various oncogenes and pathways. As a result, these insights underscore the potential of targeting METTL3 for the therapeutic intervention of BC.

### 2.2. Cross-talk between METTL3 and multi-Omics modifications: a novel epigenetic paradigm

Chen et al.^37^ found that the expression of the oncogene trophoblast cell surface protein 2 (*TROP2*) mRNA shows a positive correlation with the levels of METTL3 and the m^7^G methyltransferase METTL1 in patients with BC. METTL3-mediated m^6^A modifications on *TROP2* mRNA facilitate its translation during the malignant transformation of BC.^37^ In a similar vein, METTL1 boosts *TROP2* translation through m^7^G modifications on specific tRNAs. Furthermore, the simultaneous knockout of METTL3 and METTL1 impairs BC cell proliferation, migration, and invasion.^37^ These results reveal a novel RNA epigenetic mechanism involving METTL3 and METTL1-mediated dual m^6^A/m^7^G RNA modifications, which enhance *TROP2* translation and promote BC progression. Moreover, there is an inverse relationship between RMRP and SCARA5. The significant suppression of BC cell proliferation, migration, and invasion due to RMRP downregulation is counteracted by the overexpression of SCARA5.^38^ The interaction between METTL3-mediated m^6^A modification and DNA modification regulates this correlation. In particular, METTL3 stabilizes the mechanism where RMRP recruits DNA methyltransferases to the *SCARA5* promoter region, leading to its methylation. This epigenetic suppression of SCARA5 expression by methylation of its promoter enables METTL3 to play a promoter role in BC tumorigenesis^38^ (Figure 1, Table 1).

### 2.3. METTL3 as a proto-oncogene: diagnostic biomarker and prognostic sentinel

Elevated levels of METTL3 have been observed in BC, playing a significant role in its onset and advancement. The overproduction of METTL3 has emerged as a potential diagnostic marker for BC due to its involvement in BC formation.^45^ Further analysis has revealed a strong correlation between METTL3 and the recurrence of muscle-invasive BC, with METTL3 mutations linked to BC relapse.^39,46^ This suggests that METTL3 could serve as a prognostic marker for BC recurrence. Importantly, abnormal METTL3 expression functions as an oncogene, contributing to both the initiation and progression of BC, and holds potential as both a diagnostic and prognostic marker in clinical settings. For example, research by Wang et al.^47^ and Yang et al.^48^ has identified a significant association between METTL3 copy number variations (CNVs) and overall survival in patients with BC, reinforcing its role as an oncogene. Additionally, another study has highlighted the strong link between high METTL3 levels and poor prognosis in patients with BC.^40^ Yang et al.^40^ also found that patients with BC with METTL3 CNVs not only develop BC but also exhibit a favorable response to neoadjuvant chemotherapy, leading to significantly longer overall survival or disease- and progression-free survival compared to those with wild-type METTL3. A 2021 meta-analysis of 12 studies established METTL3 overexpression as an independent predictor of poor overall survival (pooled HR = 1.83, 95% CI: 1.45–2.30),^39^ though CNV contexts may modulate this risk. This contrasts with studies linking METTL3 overexpression to poor survival,^40,47^ suggesting tissue-specific or mutation-dependent roles. Resolving this paradox requires stratification by METTL3 mutation status in clinical cohorts. Consequently, somatic mutations in METTL3 may act as predictive markers for neoadjuvant chemotherapy response in patients with BC.

Besides its direct role in promoting cancer, METTL3 is involved in various cancer-related cellular processes such as mitotic spindle assembly, G2/M checkpoint regulation, and specific signaling pathways.^40^ For example, conditional knockout of *METTL3* in a transgenic mouse model reduces oncogenesis and tumor angiogenesis in BC stem cells (BCSCs).^34^ Mechanistically, the deletion of *METTL3* decreases the expression of tyrosine kinase endothelial (TEK) and vascular endothelial growth factor-α (VEGF-α) by reducing m^6^A peaks in specific regions. These findings collectively indicate that METTL3-mediated m^6^A modifications are crucial for TEK-VEGF-α-mediated tumor progression and angiogenesis activation. Gao et al.^32^ found that BCSCs have higher levels of global m^6^A and METTL3 expression compared to non-BCSCs. The absence of METTL3 impairs the self-renewal ability of BCSCs, as shown by reduced aldehyde dehydrogenase activity and sphere formation capacity.^32^ METTL3-mediated m^6^A modifications regulate the expression of ALF transcription elongation factor 4 (AFF4), which binds to promoter regions and maintains the transcriptional activity of SRY-box transcription factor 2 (SOX2) and c-MYC, both essential for BCSC function.^32^ Knockdown of *AFF4* mirrors the effects of *METTL3* deletion, leading to decreased tumor-initiating capability *in vivo* for BCSCs.^32^ These findings underscore the critical role of METTL3-mediated m^6^A modifications in self-renewal and tumorigenic potential through a novel signaling axis involving METTL3, AFF4, SOX2, and c-MYC. Similarly, the METTL3/AFF4 axis is implicated in human BC, with a significant upregulation of METTL3 expression. Reducing METTL3 levels diminishes the proliferation, invasion, survival, and tumorigenicity of BC cells.^33^ Conversely, increasing METTL3 expression boosts the growth and invasive potential of these cells. Comprehensive sequencing analysis identifies *AFF4*, *c-MYC*, *IKBKB* (inhibitor of NF-κB kinase subunit beta), and *RELA* proto-oncogene as direct targets of METTL3.^33^ Moreover, *AFF4* binds to the promoter region of the *c-MYC* gene to facilitate its expression. These findings reveal a novel multilevel regulatory network controlled by METTL3, involving an AFF4/NF-κB/c-MYC signaling cascade. Recently, Chen et al. (2025) identified TNF-α as a key inducer of BC metastasis via METTL3-mediated m6A modification of CLASP2 and IQGAP1 mRNAs, driving cytoskeleton remodeling. This TNF-α/METTL3/CLASP2/IQGAP1 axis represents a novel mechanotransduction pathway linking inflammation to metastatic dissemination, offering therapeutic targets for advanced BC^31^ (Figure 1, Table 1).

To conclude, these findings offer significant insights into the fundamental processes driving BC progression. They also indicate that METTL3 directly promotes oncogenic activity through various signaling pathways. Consequently, METTL3’s governance of stemness and angiogenesis informs its central role in therapy failure – a nexus explored next.

### 2.4. METTL3-driven therapeutic resistance: chemotherapy and immune evasion

Although various therapeutic approaches including chemotherapy, immune checkpoint inhibitors, and radiotherapy are available for the treatment of BC, a significant number of patients eventually experience treatment failure and succumb to drug resistance.^49^ Therefore, gaining a comprehensive understanding of the underlying mechanisms driving drug resistance in BC is crucial to enhancing the efficacy of therapy. Recent studies have highlighted the role of METTL3-mediated m^6^A modification in regulating drug resistance in BC.^50,51^

*Cisplatin* is the first-line chemotherapy for muscle-invasive and metastatic BC. However, treatment failure occurs in 60%–70% of patients due to *Cisplatin* resistance.^52,53^ Identification of key regulators that mediate *Cisplati*n sensitivity could enhance the efficacy of chemotherapy. Accumulating evidence suggests that METTL3 induces *Cisplatin* resistance in BC, indicating its potential as a crucial regulator for BC drug resistance.^54^ Wei et al.^35^ reported an association between high expression of circ0008399 and poor survival rate in patients with BC. They demonstrated that circ0008399 suppresses cell apoptosis and reduces chemosensitivity to *Cisplatin* both *in vitro* and *in vivo*. Mechanistically, circ0008399 binds with WTAP to facilitate the formation of the m^6^A methyltransferases complex (WTAP/METTL3/METTL14), thereby upregulating global m^6^A modification levels in BC cells.^35^ This promotes downstream target gene TNF alpha-induced protein 3 (TNFAIP3) expression, which positively correlates with tumorigenesis by increasing *TNFAIP3* mRNA stability through an m^6^A-dependent mechanism.^35^ TNFAIP3 acts as an anti-apoptotic protein inhibiting TNF-induced apoptosis by decreasing caspase-8 activity and also functions as an NF-kB-responsive gene that deactivates the sustained NF-kB pathway.^35^ In conclusion, the circ0008399/m^6^A methyltransferases complex/TNFAIP3 pathway contributes to reduced chemosensitivity of BC to *Cisplatin* by preventing apoptosis through the inhibition of the NF-kB pathway. These results underscore the significance of the m^6^A methyltransferases complex in modulating BC’s responsiveness to *Cisplatin* and propose that targeting the circ0008399/m^6^A methyltransferases complex/TNFAIP3 pathway could be a promising approach to enhance BC’s sensitivity to *Cisplatin* chemotherapy. Per EAU/NCCN guidelines,^19,49^ cisplatin failure in metastatic BC necessitates biomarker-driven alternatives. METTL3‘s dual role in chemoresistance (circ0008399/TNFAIP3) and immune evasion (PD-L1) positions it as a priority target in consensus algorithms.

*Melittin* holds significant potential as a therapeutic agent for BC.^55^ Research by Yan et al.^56^ revealed that *Melittin’s* anti-tumor effects are linked to METTL3-mediated m^6^A modification. *Melitti*n specifically targets METTL3, disrupting miR-146a-5p maturation and activating the NUMB/NOTCH2 axis in BC cells. As a result, higher levels of METTL3 and miR-146a-5p are associated with recurrence and poor prognosis in patients with BC. The METTL3/miR-146a-5p pathway’s dysregulation plays a crucial role in BC progression. Importantly, METTL3 determines BC cells’ sensitivity to *Melittin*, emphasizing the oncogenic role of the METTL3/miR-146a-5p/NUMB/NOTCH2 axis and its potential as a target for treating recurrent BC.

### 2.5. METTL3 as therapeutic vulnerability: overcoming chemoresistance and immune escape

Beyond chemoresistance, METTL3 concurrently subverts immune surveillance – a dual barrier to treatment efficacy. We synthesize these interconnected vulnerabilities below. Recent decades have seen remarkable progress in cancer immunotherapy, with checkpoint inhibitors being used as first- or second-line treatments for patients with BC.^57–59^ Ni et al.^36^ have shown that the activated JNK signaling pathway is closely linked to the abnormal expression of METTL3. Suppressing JNK1 hinders c-Jun’s ability to bind to the *METTL3* promoter, thereby reducing METTL3 expression and global m^6^A levels. Mechanistically, METTL3/m^6^A modification occurs on the 3’-UTR region of *PD-L1* mRNA, recognized by IGF2BP1 to stabilize *PD-L1* mRNA and enhance its expression. NCCN-endorsed biomarkers for BC immunotherapy resistance (e.g., TMB, PD-L1) now warrant expansion to include METTL3,^49^ given its JNK-dependent regulation of PD-L1 aligns with consensus pathways. PD-L1, a co-inhibitory immune checkpoint protein, is overexpressed not only in cancer cells but also in various cell types within the tumor microenvironment.^60^ These observations indicate that JNK signaling aids immune evasion in BC through a METTL3-dependent mechanism. Thus, inhibiting JNK reduces METTL3 expression, subsequently making BC cells more susceptible to CD8^+^ T cell cytotoxicity by regulating PD-L1 expression. Therefore, targeting the JNK1/METTL3 axis could be a promising strategy for modulating immune therapy in BC. Wu et al. (2023) revealed that PD1hiCD200hiCD4+ exhausted T cells promote EMT and immunotherapy resistance in BC. This aligns with METTL3-mediated PD-L1 stabilization, suggesting that combinatorial targeting of METTL3 and T-cell exhaustion markers (e.g., anti-CD200) may overcome immune evasion.^61^

*Cisplatin*, a first-line drug for BC, demonstrates its tumor-inhibitory effects and therapeutic role in preventing metastasis through METTL3-mediated m^6^A modification.^62^ Specifically, *Cisplatin* administration significantly reduces METTL3 expression, which is deemed a risk factor for BC.^63^ The functional mechanism of *Cisplatin* involves regulating granulocyte colony-stimulating factor (G-CSF) via METTL3-mediated m^6^A modification. G-CSF is a vital cytokine mediating the immunomodulatory effects induced by *Cisplatin*, produced by fibrocystic bone marrow-derived suppressor cells, leading to a significant stimulation of leukocytes and a leukemia reaction.^63,64^ Collectively, these findings suggest that *Cisplatin* decreases the number of fibrocystic bone marrow-derived suppressor cells by inhibiting G-CSF methylation through targeting METTL3.

## 3. Discussion-METTL3 in BC: bridging mechanistic insights to clinical translation

Our analysis validates METTL3‘s dual role: as an oncogenic driver (evidenced by HIF1A/BIRC5, AFF4/c-MYC axes), and as a therapeutic vulnerability (circ0008399/TNFAIP3 chemoresistance reversal; JNK/PD-L1 immune sensitization). However, context-dependent contradictions—e.g., METTL3 CNVs correlating with both poor prognosis^47^ and chemotherapy response^40^—warrant subtype-specific biomarker validation. BC is marked by high rates of recurrence and treatment failures in clinical practice, posing a significant threat to human health due to its unclear pathogenesis. Therefore, it is crucial to explore the mechanisms underlying the onset, progression, metastasis, and drug resistance of BC, which has attracted substantial attention. Specifically, numerous studies have addressed these issues from the innovative perspective of METTL3-mediated m^6^A modification. However, no existing literature has provided a comprehensive review of these investigations. Consequently, our goal is to offer a review that thoroughly interprets these studies and serves as a valuable resource for researchers.

In this review, we emphasize the pivotal role of METTL3 in the development and progression of BC. METTL3 influences BC growth, malignancy, and response to chemotherapeutic agents by directly or indirectly regulating specific genes. The mechanism through which METTL3 operates in BC involves modulating proliferation, migration, invasion, and EMT in BC cells. Additionally, METTL3 is involved in various cancer-related cellular processes, such as mitotic spindle assembly, G2/M checkpoint control, and targeted signaling pathways. Together, these findings indicate that METTL3 exerts both direct and indirect oncogenic effects on BC through multiple signaling pathways. Thus, we propose METTL3 as a potential biomarker for diagnosing and prognosticating BC. Moreover, targeting METTL3 offers therapeutic potential for BC by reducing immunotherapy resistance and enhancing *Cisplatin* sensitivity, suggesting its viability as a treatment target.

## 4. Uncharted territories: bridging mechanistic duality to clinical translation

Furthermore, this review highlights the limited current research on METTL3 modifications in BC. This gap in literature presents a significant opportunity for future investigations, particularly given the crucial role that mRNA methylation plays in regulating gene expression and cellular processes. The understudied nature of METTL3 in this context suggests a need for comprehensive studies that not only explore its functional implications but also unravel the molecular mechanisms through which METTL3 contributes to BC pathogenesis. To better understand the relevance of METTL3 in BC, it is essential to consider the broader landscape of RNA methylation and its implications in oncogenesis.

Despite promising preclinical data, clinical translation faces four key challenges^1^: Target Selectivity: METTL3 inhibitors (e.g., STM2457) risk disrupting physiological m^6^ A functions in non-target tissues due to ubiquitous RNA methylation roles. Tumor-specific delivery remains critical.^2^ Drug Delivery: Nucleic acid-based therapies (METTL3-siRNA) require advanced nanocarriers (e.g., EGFR-antibody-conjugated nanoparticles) for bladder-specific targeting, yet in vivo stability and penetration barriers persist.^3^ Compensatory Resistance: Demethylase upregulation (ALKBH5/FTO) may restore m^6^ A levels post-METTL3 inhibition, necessitating combination regimens with eraser inhibitors.^4^ Clinical Validation: No METTL3-targeted trials exist for BC; phase I studies exploring METTL3i + anti-PD-1 (e.g., NCT054XXXX) are urgently needed to validate synergy. Emerging evidence suggests that METTL3-mediated m^6^A modification can influence various aspects of tumor biology, including cell proliferation, apoptosis, and metastasis. For instance, studies in other cancer types have demonstrated that altered levels of METTL3 can lead to significant changes in the stability and translation of oncogenes and tumor suppressors. This raises critical questions about how similar mechanisms might operate in BC and whether METTL3 could serve as a potential therapeutic target.

### 4.1. Clinical translation roadmap

To accelerate the clinical application of METTL3 targeting, we propose a prioritized, phased approach^1^: Phase 0 (Preclinical Optimization): Focus on developing bladder tumor-specific METTL3 inhibitor delivery systems. Strategies include nanoparticle conjugation (e.g., EGFR-antibody-conjugated nanoparticles for selective delivery of compounds like STM2457) to minimize off-target effects on physiological RNA methylation.^2^ Phase I (Early Clinical Trials): Initiate clinical trials evaluating the safety and preliminary efficacy of combining METTL3 inhibitors (METTL3i) with existing immunotherapies, particularly anti-PD-1/PD-L1 agents (e.g., protocol NCT054XXXX), in patient cohorts with cisplatin-resistant metastatic BC.^3^ Phase II (Biomarker-Driven Validation): Prospectively validate METTL3 copy number variations (CNVs) and overexpression levels as predictive biomarkers for response to neoadjuvant chemotherapy and immunotherapy across BC molecular subtypes.

### 4.2. Unresolved mechanistic questions

Beyond translational efforts, critical mechanistic gaps demand resolution^1^: How do somatic mutations or post-translational modifications of METTL3 alter its substrate specificity, methyltransferase activity, and interaction partners within different BC subtypes (e.g., luminal vs. basal)?^2^Does pharmacological inhibition of METTL3 synergize with therapies targeting complementary epigenetic pathways, such as DNA methyltransferase inhibitors (e.g., decitabine) or histone deacetylase (HDAC) inhibitors, to overcome compensatory resistance mechanisms involving demethylases (ALKBH5/FTO)?^3^What is the precise interplay between METTL3-mediated m^6^ A modification and other RNA modifications (e.g., m^5^ C, Ψ) in regulating oncogenic networks specific to BC progression and therapy resistance?

Moreover, the interplay between METTL3 and other epitranscriptomic modifiers, such as the demethylase FTO or the reader proteins YTHDF1, YTHDF2, and YTHDC1, warrants deeper exploration. Understanding these interactions could provide insights into the regulatory networks governing BC progression. For example, the cooperative or antagonistic effects of these modifiers on mRNA metabolism might influence the expression of key genes involved in BC, ultimately affecting tumor behavior and patient outcomes. In addition to molecular mechanisms, it is crucial to investigate the potential clinical implications of METTL3 in BC. Biomarker studies could focus on the expression levels of METTL3 and its downstream targets in patient samples, correlating these findings with clinical outcomes such as response to therapy or overall survival. Such investigations could pave the way for the development of novel prognostic tools and therapeutic strategies that leverage the modulation of METTL3 activity. Furthermore, the integration of METTL3 research with advanced techniques such as CRISPR-Cas9 gene editing or RNA sequencing could facilitate a more nuanced understanding of its role in BC. By employing these technologies, researchers could dissect the functional consequences of METTL3 knockout or overexpression in BC cell lines and animal models. This experimental approach would allow for the identification of specific pathways and targets that are influenced by METTL3, offering a pathway for the development of targeted therapies.

In summary, although the existing research on METTL3 in BC is still in its infancy, there is significant potential for meaningful discoveries. Future investigations should focus on clarifying the diverse roles that METTL3 plays in BC biology, delving into its molecular mechanisms as well as its clinical implications. By addressing these gaps in knowledge, researchers can enhance our overall understanding of BC and may uncover new therapeutic strategies that could lead to improved patient outcomes. This quest for knowledge not only promises to propel BC research forward but also highlights the critical role of RNA modifications in the broader context of cancer biology.

## 5. Conclusion-METTL3: a beacon for precision oncology in BC

Overall, this review advances our understanding of the relationship between METTL3 and BC, positioning METTL3 as a promising diagnostic biomarker and actionable target.

## Supplementary Material

Highlights_KCBT-S-2025-0229.R2.doc

Figure legends.doc

## Data Availability

Data sharing is not applicable to this article as no datasets were generated or analyzed during the current study.

## Highlights

- METTL3 is a risk gene for BC pathogenesis
- METTL3 contributes to BC onset and malignant progression
- METTL3 relates to BC metastasis and drug resistance
- Downregulating METTL3 is effective for BC treatment
- METTL3 with the potential to diagnose and treat BC

## List of abbreviations

BC: Bladder cancer
METTL3: Methyltransferase-like 3
m^6^A: N^6^-methyladenosine
EMT: epithelial-mesenchymal transition
IGF2BP: Insulin-like growth factor 2 mRNA-binding proteins
VEGF-α: Vascular endothelial growth factor-α
PTEN: Phosphatase and tensin homolog
P3H4: Prolyl 3-hydroxylase family member 4
ITGA6: Integrin subunit alpha 6
BLACAT3: BLC-associated transcript 3
TROP2: Trophoblast cell surface protein 2
SETD7: SET domain-containing 7
KLF4: Krüppel-like factor 4
CNVs: Copy number variation
AFF4: ALF transcription elongation factor 4
SOX2: SRY-box transcription factor 2
c-MYC: Cellular MYC
TEK: Tyrosine endothelial kinase
IKBKB: Inhibitor of NF-κB
RELA: RELA proto-oncogene
G-CSF: Colony-stimulating factor.
