# Supplementary material for: Unraveling the dual role of METTL3-mediated m6A RNA modification in bladder cancer: mechanisms, therapeutic vulnerabilities, and clinical implications
Source: Cancer Biol Ther. 2025 Aug 8;26(1):2545057. doi: 10.1080/15384047.2025.2545057 (PMC12919897; doi:10.1080/15384047.2025.2545057)
Supplement: Highlights_KCBT-S-2025-0229.R2.doc [file KCBT_A_2545057_SM6029.doc]

**Unraveling the Dual Role of METTL3-Mediated m^6^A RNA Modification in Bladder Cancer: Mechanisms, Therapeutic Vulnerabilities, and Clinical Implications**

Hua Chun^1^, Kangzhuo Baima^1*^

^1^College of Medicine, Tibet University, Tibet 850000, China

***Correspondence:**

Kangzhuo Baima

E-mail: tibet2025@163.com

***Running title:*** *Functions and potentials of METTL3 in Bladder Cancer*

**Highlights:**

1. METTL3 is a risk gene for BC pathogenesis

2. METTL3 contributes to BC onset and malignant progression

3. METTL3 relates to BC metastasis and drug resistance

4. Downregulating METTL3 is effective for BC treatment

5. METTL3 with the potential to diagnose and treat BC
