# Supplementary material for: Unraveling the dual role of METTL3-mediated m6A RNA modification in bladder cancer: mechanisms, therapeutic vulnerabilities, and clinical implications
Source: Cancer Biol Ther. 2025 Aug 8;26(1):2545057. doi: 10.1080/15384047.2025.2545057 (PMC12919897; doi:10.1080/15384047.2025.2545057)
Supplement: Figure legends.doc [file KCBT_A_2545057_SM6028.doc]

**Figure legends**

**Figure 1 METTL3-centric network driving BC pathogenesis**

As the core m⁶A methyltransferase, METTL3 orchestrates BC pathogenesis by dynamically regulating RNA stability, translation, and degradation differently. This figure integrates molecular mechanisms of METTL3’s function in BC, highlighting METTL3 as a disease driver in BC. Arrows denote activation; blunt lines inhibition.

METTL3, Methyltransferase-like 3; BC, bladder cancer; m^6^A, N^6^-methyladenosine; IGF2BP, Insulin-like growth factor 2 mRNA-binding proteins; VEGF-α, Vascular endothelial growth factor-α; PTEN, Phosphatase and tensin homolog; P3H4, Prolyl 3-hydroxylase family member 4; ITGA6, Integrin subunit alpha 6; BLACAT3, BLC-associated transcript 3; TROP2, Trophoblast cell surface protein 2; SETD7, SET domain-containing 7; KLF4, Krüppel-like factor 4; CNVs, Copy number variation; AFF4, ALF transcription elongation factor 4; SOX2, SRY-box transcription factor 2; c-MYC, Cellular MYC; TEK, Tyrosine endothelial kinase; IKBKB, Inhibitor of NF-κB; RELA, RELA proto-oncogene; EMT, epithelial-mesenchymal transition.
